# Antiphospholipid syndrome in 2014: more clinical manifestations, novel pathogenic players and emerging biomarkers

**DOI:** 10.1186/ar4549

**Published:** 2014-04-23

**Authors:** Pier Luigi Meroni, Cecilia Beatrice Chighizola, Francesca Rovelli, Maria Gerosa

**Affiliations:** 1 Division of Rheumatology - Istituto Ortopedico Gaetano Pini, Department of Clinical Sciences and Community Health, University of Milan, 20122 Milan, Italy; 2 Istituto Auxologico Italiano, via Zucchi 18, 20095 Cusano Milanino, Mi, Italy

## Abstract

The clinical spectrum of the anti-phospholipid syndrome (APS) is not limited to vascular thrombosis or miscarriages but includes additional manifestations that cannot be explained solely by a thrombophilic state. Anti-cardiolipin, anti-beta_2_ glycoprotein I (anti-β_2_GPI) and lupus anticoagulant (LA) assays are not only the formal diagnostic and classification laboratory tools but also parameters to stratify the risk to develop the clinical manifestations of the syndrome. In particular, anti-β_2_GPI antibodies reacting with an immunodominant epitope on domain I of the molecule were reported as the prevalent specificity in APS patients, correlating with a more aggressive clinical picture. Several laboratory assays to improve the diagnostic and predictive power of the standard tests have been proposed. Plates coated with the phosphatidylserine-prothrombin complex for detecting antibodies represent a promising laboratory tool correlating with LA and with clinical manifestations. Anti-phospholipid antibodies can be found in patients with full-blown APS, in those with thrombotic events or obstetric complications only or in asymptomatic carriers. An inflammatory second hit is required to increase the presence of β_2_GPI in vascular tissues, eventually triggering thrombosis. Post-transcriptional modifications of circulating β_2_GPI, different epitope specificities or diverse anti-β_2_GPI antibody-induced cell signaling have all been suggested to affect the clinical manifestations and/or to modulate their occurrence.

## Review

## The current clinical spectrum of anti-phospholipid syndrome

### Formal clinical classification criteria

The revised classification criteria for anti-phospholipid syndrome (APS), commonly used as a diagnostic tools for the syndrome, include a history of venous or arterial thrombosis and/or of pregnancy morbidity in the presence of persistent anti-phospholipid antibody (aPL) positivity (Table 1) [1]. The most common vascular manifestations are deep venous thrombosis of lower limbs, pulmonary embolism and cerebral ischemic attack; early and late miscarriages are the major features of obstetric APS [1,2]. In the catastrophic variant of APS, multiple small-vessel thrombotic events occur at different sites, in association with manifestations of the systemic inflammatory response syndrome [3].

**Table 1:** **Revised classification criteria for anti-phospholipid syndrome**[1]

**Clinical criteria**	
Vascular thrombosis	One or more episodes of arterial, venous or small vessel thrombosis in any tissue or organ (confirmed by objective validated criteria (imaging study or histopathology))
Pregnancy complications	One or more unexplained deaths of a morphologically normal fetus ≥10th gestational week
	One or more premature births (≤34th gestational week) of a morphologically normal neonate because of eclampsia, severe pre-eclampsia or placental insufficiency
	Three or more unexplained consecutive spontaneous abortions ≤9th gestational week (maternal anatomic and hormonal abnormalities and chromosomal abnormalities excluded)
**Laboratory criteria**	
Lupus anticoagulant present in plasma	Detected according to the guidelines of the International Society of Thrombosis and Haemostasis (Scientific subcommittee on lupus anticoagulant/phospholipid-dependent antibodies)
IgG and/or IgM anti-cardiolipin antibodies in serum of plasma	At medium/high titer (≥40 GPL or MPL or ≥99th percentile) measured by standard ELISA
IgG and/or IgM anti-β_2_ glycoprotein I antibodies in serum or plasma	Titer ≥99th percentile measured by standard ELISA, according to recommended procedures

At least one clinical and one laboratory criterion is mandatory. Autoantibodies have to be confirmed on two or more occasions at least 12 weeks apart. ELISA, enzyme-linked immunosorbent assay; GPL, IgG aPL units; Ig, immunoglobulin; MPL, IgM aPL units.

### Clinical manifestations not yet considered as classification criteria

From the first definition of the disease, the clinical spectrum of APS has notably extended and many other manifestations have been described [4]. Thrombocytopenia, heart valve disease (valve thickening, vegetations and regurgitation), nephropathy, livedo reticularis and skin ulcers are relatively common features of APS but are not included in the classification criteria because of their low specificity (Table 2) [5-9].

**Table 2:** Anti-phospholipid syndrome clinical manifestations not yet considered as classification criteria

	**Frequency**		
**Clinical manifestations**	**PAPS**	**APS-SLE**	**Notes**
Thrombocytopenia	20-25%	30-40%	Usually mild
			No protective effect on thrombotic risk
Heart valve disease	12-33%	40%	Possibly an additional risk for secondary thromboembolism
Skin			
Livedo reticularis	20-25%	35%	Original association with arterial thrombosis not confirmed in prospective studies
Ulcers	33%	7-10%	Pre-tibial area
			Frequently observed in catastrophic APS
Superficial thrombophlebitis	9%		Reported in aPL-positive patients but their value still debated
Kidney			
Renal artery stenosis	26% of aPL + patients with uncontrolled hypertension		Resulting in severe renovascular hypertension, renal infarcts
APS nephropathy (renal small artery vasculopathy, involving both arterioles and glomerular capillaries)	35%^a^	39-67% ^a^	Association with pregnancy complications, extra-renal vascular thrombosis and higher risk of chronic renal failure among SLE patients
Central nervous system			
Migraine/headache	20%	25%	Controversial association with aPLs because of the high prevalence in the general population
Epilepsy	6-7%	14%	In many but not all cases secondary to ischemic events
			Conflicting data on relationship between aPLs and seizure in SLE
MS-like disease			No definite data regarding prevalence because of the difficult differential diagnosis
Cognitive impairment	38%	48%	Mostly involving attention and verbal fluency
Dementia	2.5-56%		Resulting from chronic or recurrent ischemic events
Ocular manifestations	15-88%		Amaurosis fugax as potential first sign of cerebral ischemia
			Retinal artery thrombosis vessels (arteries and veins) are relatively frequent and can lead to significant visual loss
Transverse myelopathy	1%		Strong correlation with aPLs in SLE patients
Pulmonary alveolar hemorrhage	<1%		Very poor prognosis

^a^Data from small series, with hypertension or signs suggestive of nephropathy. aPL, anti-phospholipid antibody; APS, anti-phospholipid syndrome; MS, multiple sclerosis; PAPS, primary anti-phospholipid syndrome; SLE, systemic lupus erythematosus.

In addition to thrombo-occlusive events in the cerebral circulation, a wide range of 'non-criteria' neurological manifestations have been associated with aPL, even though in some cases such association is still controversial. Examples include untreatable headache and migraine, epilepsy, chorea, ocular manifestations such as amaurosis fugax and retinal vessel thrombosis [10,11]. A clinical syndrome and/or magnetic resonance imaging resembling multiple sclerosis has also been described in APS, raising the issue of a correct differential diagnosis [10].

Persistent aPL positivity has been related to cognitive impairment in systemic lupus erythematosus (SLE) patients, even if recent studies in very large SLE cohorts have not confirmed such an association [11]. Although scant, studies in primary APS have reported a high predominance of cognitive deficits involving attention and verbal fluency [12,13]. In addition, several authors reported the occurrence of chronic or recurrent ischemic events affecting small or large cerebral vessels and leading to multi-infarct dementia [1].

Further manifestations affecting different organs or tissues have been described in APS patients. However, their association with aPL is still a matter of research mainly because of the anecdotal nature of the reports or the presence of other underlying disorders (diffuse alveolar hemorrhage, myocardial dysfunction, transverse myelopathy, Guillain-Barré syndrome and multiple mononeuropathy, sensorineural hearing loss or vertigo due to middle ear involvement, splinter hemorrhages and anetoderma [1]).

## Diagnostic laboratory tools

### Classification laboratory assays

Laboratory criteria for formal APS classification currently include three aPL assays: one based on coagulation tests to reveal the presence of lupus anticoagulant (LA) and two solid phase assays to detect IgG/IgM antibodies targeting cardiolipin (CL)/beta_2_ glycoprotein I (β_2_GPI) complexes or β_2_GPI alone (Table 1). Persistent medium/high positivity (12 weeks apart) of at least one of these tests is mandatory [1].

### Risk stratification

According to the revised classification criteria, APS patients should be divided into four categories: category I includes patients with more than one positive test in any combination, while patients with a single positive test should be classified in category II (IIA if LA-positive, IIb if positive for antibodies against CL (aCLs), IIc if positive for anti-β_2_GPI antibodies) [1]. Triple positivity, defined by the presence of LA and medium/high titers of aCL and anti-β_2_GPI antibodies (above the 99th percentile), is the most predictive profile for clinical manifestations and recurrences despite conventional treatment [14,15].

There is growing evidence that patients in category II have a lesser risk to develop APS manifestations. LA was reported to be the most predictive test. LA can be mediated by both anti-β_2_GPI and anti-prothrombin (aPT) antibodies [16]. However, β_2_GPI-dependent LA was found to be a stronger risk factor for thrombosis and miscarriages than aPT-dependent LA [17,18]. aCL positivity alone is not associated with an increased risk of thrombosis or pregnancy loss [16,17,19]. Data on anti-β_2_GPI antibodies are more controversial, maybe because the assay is less standardized [17]. Accordingly, Otomo and colleagues [20] have recently validated a scoring system to quantify the thrombotic or obstetric risk depending on aPL profiles.

Clinical events are more robustly associated with aPLs of the IgG isotype, an isolated positivity for aCLs or anti-β_2_GPI antibodies of the IgM isotype being rarely detected in APS cohorts. Patients carrying both aCL/anti-β_2_GPI antibody isotypes display a higher risk of developing clinical events [21]. Some investigators have recently proposed that aCLs and anti-β_2_GPI IgA antibodies be included in the APS laboratory criteria. However, this is not supported by available data: the detection of a single IgA aPL positivity is more commonly associated with non-criteria manifestations, while IgA testing has not been shown to increase the diagnostic accuracy for APS [22]. Interestingly, this is not consistent with *in vivo* findings, which are supportive for a pathogenic role of IgA β_2_GPI-dependent aPLs in mediating thrombus formation [23].

While it is well accepted that aPLs confer a prothrombotic susceptibility when at high titers, controversies have recently arisen about the clinical meaning of low-titer aPLs in pregnancy morbidity. A few studies have reported that women with persistent low-titer aPL positivity display an obstetric outcome comparable to the general population [17]. On the other hand, a recent study showed that low-titer aCL and anti-β_2_GPI antibody positivity (between the 95th and 99th percentiles) accurately identifies women with aPL-related pregnancy complications [24].

Risk stratification for thrombotic events should also take into account the presence of traditional cardiovascular factors such as systemic inflammatory conditions (infectious or autoimmune), inherited thrombophilia, arterial hypertension, cigarette smoking and dyslipidemia [1]. Risk-factors for pregnancy failure include low complement levels, decreased platelet counts and a previous history of thrombosis and pregnancy failure [15].

## Non-classification laboratory assays

Additional laboratory tests to detect aPLs have been reported: the most important 'non-classification' tests still deal with the two major phospholipid (PL)-binding proteins thought to represent the true antigenic targets for aPL: β_2_GPI and prothrombin (PT) [16]. However, other autoantigens have been described that are a matter of debate and research (Table 3).

**Table 3:** Future research requirements for the most promising non-classification laboratory assays

**Test**	**Assay**	**Future research needs**
Anti-DI antibodies	ELISA	Analytical and post-analytical standardization
	CIA	Retrospective and prospective clinical validation
Anti-PS/PT antibodies	ELISA	Analytical and post-analytical standardization
		Retrospective confirmatory studies and prospective clinical validation
Anti-PE antibodies	ELISA	Analytical and post-analytical standardization
		Retrospective confirmatory studies and prospective clinical validation
Annexin A5 resistance assay	Two-step coagulation assay	Analytical and post-analytical standardization
		Retrospective and prospective clinical validation

CIA, chemiluminescence immunoassay; DI, domain I; ELISA, enzyme-linked immunosorbent assay; PE, phosphatidylethanolamine; PS/PT, phosphatidylserine-prothrombin.

### Anti-prothrombin antibodies

To be antigenically recognized, human PT has to be either coated on activated plates or exposed to immobilized anionic phosphatidylserine (PS) via calcium ions. ELISAs to detect antibodies against the PS/PT complex (aPS/PT antibodies) identify a partially different autoantibody population from the assay using PT as the only antigen [25]. A contentious issue concerns the potential cross-reactivity between aPS/PT and anti-β_2_GPI antibodies; however, human anti-β_2_GPI monoclonals or affinity-purified anti-β_2_GPI polyclonal IgG antibodies obtained from a serum reacting with both β_2_GPI and PS/PT have been shown to react towards β_2_GPI only [26].

*In vitro* experimental findings suggest that aPTs exert thrombogenic effects interfering with fluid phase components of coagulation and activating endothelial cells (ECs). Evidence from animal models is rather weak, however, mainly because of the lack of cross-reactivity of human aPTs with animal PT [25].

The wide variability in epitope specificities and detection methods drives a disparity across available studies about the prevalence and clinical significance of aPTs. The prevalence of antibodies targeting PT depends also on selection of study populations: when considering solely individuals with LA, the positivity rate increases up to 85% and 88% for aPTs and aPS/PTs, respectively [25]. Similarly, the clinical significance of aPTs in both primary and secondary APS is still a matter of debate. Some studies showed that aPTs are an independent risk factor for either venous or arterial thrombosis, while others have failed to demonstrate such an association. On the other hand, most of the studies addressing the clinical significance of aPS/PTs have highlighted a significant association with aPL-associated manifestations, in particular venous thrombosis. Consistently, a systematic review did not find any correlation between aPTs and clinical events, while a more recent one found that aPS/PTs are a stronger risk factor for arterial and venous thrombosis than aPTs [27]. Much more controversial remains the association of aPTs and aPL-related pregnancy morbidity (Tables 4 and 5).

**Table 4:** Studies addressing prevalence and clinical association of aPT antibodies

**Reference**	**Study population**	**N**	**Prevalence of anti-PT**	**Clinical association**
Fleck *et al*. [28]	LA positive subjects	42	74%	NI
Pengo *et al*. [29]	APS patients	22	50%	No association with thrombosis
Horback *et al*. [30]	SLE patients	175	38%	Association with thrombosis (IgG and IgM)
Puurunen *et al*. [31]	SLE patients	139	34%	Association with DVT
Swadzba *et al*. [32]	SLE patients with thrombotic event	127	28%	No association with thrombosis (IgG and IgM)
		31		
Galli *et al*. [33]	aPL-positive subjects	59	58%	No association with thrombosis
			IgG 35.6%	
			IgM 37.3%	
Bertolaccini *et al*. [34]	SLE patients	207	28%	No association with APS clinical manifestations
Forastiero *et al*. [35]	APS patients	97	25%	Association with thrombosis
	aPL-negative patients with thrombotic events	83		
Munoz-Rodriguez *et al*. [36]	APS patients	70	57%	Association with arterial thrombosis (IgG only)
	SLE patients	107	40%	
Atsumi *et al*. [37]	Patients with autoimmune diseases	265	IgG: PAPS 15%; SLE APS 42%; SLE no APS 20%	No association with APS
			IgM: PAPS 5%; SLE APS 4%; SLE No APS 6%	
Galli *et al*. [38]	LA-positive patients	72	85%	No association with APS
Nojima *et al*. [39]	SLE patients	124	IgG 52.4%	Association with venous thromboembolism (only aPT IgG + LA)
			IgM 21%	
Nojima *et al*. [40]	SLE patients	168	56%	Association with arterial thrombosis
Simmelink *et al*. [41]	LA-positive patients	46	30%	Association with thrombosis
	LA-positive patients with SLE	29	LA-positive subjects: 70%	
	LA-negative patients	38		
	LA-negative patients with SLE	36		
Salcido-Ochoa *et al*. [42]	APS patients	38	IgG 26%, IgM 11%	Association with thrombosis
	SLE patients	466	IgG 20%, IgM 33%	
Von Landenberg *et al*. [43]	APS patients	170	IgG 61.7%	Association with pregnancy loss (IgG only)
	(57% PAPS; 43% SAPS)		IgM 57.6%	
			IgA 7%	
Musial *et al*. [44]	APS patients	22	IgG 45.4%, IgM 50%	No association with thrombosis
	SLE patients	160	IgG 18.1%, IgM 18.7%	
	SLE-like patients	22	IgG 31.8%, IgM 27.3%	
Ishikura *et al*. [45]	SLE patients	22	18.2%	Association with venous thrombosis
	Patients with			
	DVT/PTE	48	IgG 29%, IgM 8.3%	
	Stroke	30	IgG 16.7%, IgM 6.7%	
Koskenmies *et al*. [46]	SLE patients	292	20%	Association with arterial thrombosis
Bertolaccini *et al*. [47]	SLE patients	212	31%	Association with venous/arterial thrombosis (IgG only)
			IgG-only 24.5%	
			IgM-only 5%	
Bizzarro *et al*. [48]	aCL-positive APS patients	25	60%	Association with thrombosis (IgG only)
	SLE-APS patients	23	45%	
	SLE-no APS patients	66		
Forastiero *et al*. [49]	aPL-positive subjects (LA/aCL)	194	46%	Association with thrombosis (IgG only)
			IgG 36%	
			IgM 23%	
Gould *et al*. [50]	SLE patients	100	20%	No association with thrombosis
Tsutumi *et al*. [51]	SLE patients	139	25%	Association with thrombosis
Nojima *et al*. [52]	SLE patients	175	54.3%	No association with thrombosis
Bizzaro *et al*. [53]	SLE patients	101	IgG 13.9%	Association with thrombosis (IgG only)
			IgM 9%	
			IgG + IgM 3%	
Sailer *et al*. [54]	LA-positive subjects	79		No association with thrombosis
	With thrombosis	50	72% (assay I), 50% (assay II)	
	Without thrombosis	29	66% (assay I), 41% (assay II)	
Bardin *et al*. [55]	APS patients	62	42%	NI
Jakowski *et al*. [56]	APS patients	58	22%	No association with pregnancy loss
	Women with recurrent pregnancy loss	66	12%	
Szodoray *et al*. [57]	SLE patients	85	IgG 18%, IgM 0	NI
Pengo *et al*. [58]	LA-positive subjects	231	IgG 26%	No association with APS clinical events
			IgM 27%	
Marozio *et al*. [59]	Obstetric APS patients	187	29.4%	Association with severe pre-eclampsia, HELLP syndrome, intra-uterine fetal death
			IgG 25.8%	
			IgM 1.8%	
			IgG + IgM: 1.8%	
Hoxha *et al*. [60]	PAPS patients	158	IgG 23.5%, IgM 4.9%	Association with thrombosis and obstetric manifestations (IgG only)
	Thrombotic APS	56	IgG 10.7%, IgM 1.8%	
	Obstetric APS	102		
Sater *et al*. [61]	Women with recurrent miscarriages	277	IgM 12%	No association with pregnancy loss

aCL, anti-cardiolipin antibody; aPL, anti-phospholipid antibody; APS, anti-phospholipid syndrome; aPT, anti-prothrombin antibody; DVT, deep vein thrombosis; HELLP, hemolysis, elevated liver enzymes and low platelet count; Ig, immunoglobulin; LA, lupus anticoagulant; NI, not investigated; PAPS, primary anti-phospholipid syndrome; PTE, pulmonary thromboembolism; SAPS, secondary anti-phospholipid syndrome; SLE, systemic lupus erythematosus.

**Table 5:** Studies addressing prevalence and clinical association of aPS/PT antibodies

**Reference**	**Studied population**	**N**	**Prevalence of anti-PS/PT**	**Clinical association**
Galli *et al*. [33]	aPL-positive subjects	59	90%	No association with thrombosis
			IgG 75%	
			IgM 66%	
Atsumi *et al*. [37]	Patients with autoimmune diseases	265	IgG: PAPS 19%; SLE APS 63%; SLE-no APS 13%	Association with APS
			IgM: PAPS 10%; SLE APS 29%; SLE-no APS 4%	
Nojima *et al*. [62]	SLE patients	126	38.1%	No association with stroke
Bertolaccini *et al*. [47]	SLE patients	212	31%	No association with thrombosis
			IgG-only 16%	
			IgM-only 6%	
Tsutumi *et al*. [51]	SLE patients	139	21%	Association with thrombosis
Nojima *et al*. [52]	SLE patients	175	43.4%	Association with thrombosis
Bardin *et al*. [55]	APS patients	62	55%	NI
Jakowski [56]	APS patients	58	44%	No association with pregnancy loss
	Women with RPL	66	1%	
Atsumi *et al*. [63]	Patients with autoimmune diseases	441	18.3%	Association with APS
	PAPS	84	39%	
	SLE-APS	68	47%	
	SLE-no APS	136	10%	
	Rheumatoid arthritis	46	0	
	Sjogren syndrome	36	0	
	Other	71	4%	
Žigon *et al*. [64]	APS patients	100	59%	NI
Vlagea *et al*. [65]	PAPS patients	98	51%, IgG 35.7%, IgM 32.6%	Association with venous thrombosis and obstetric morbidity
	SAPS patients	45	53.3%, IgG 40%, IgM 31.1%	
	aPL-positive subjects	57	38.6%, IgG 21.1%, IgM 26.3%	
Pregnolato *et al*. [26]	APS patients	80	81.3%	Association with venous thrombosis (IgG only)

aPL, anti-phospholipid antibody; APS, anti-phospholipid syndrome; aPS, anti-phosphatidylserine antibodies; Ig, immunoglobulin; NI, not investigated; PAPS, primary anti-phospholipid syndrome; PS, phosphatidylserine; PT, prothrombin; RPL, recurrent pregnancy loss; SAPS, secondary anti-phospholipid syndrome; SLE, systemic lupus erythematosus.

aPS/PTs have been proposed as a surrogate test for LA and as an additional serological marker of APS, to be performed with other aPL tests to improve diagnosis. Noteworthy, LA together with aβ_2_GPI and aPS/PT antibodies has recently been identified to display the best diagnostic accuracy for both vascular and obstetric APS among 23 possible combinations of six aPL assays (LA, aCLs, anti-β2GPI antibodies, aPTs, aPS/PTs, and anti-phosphatidylethanolamine antibodies (aPEs)) [66]. However, the bulk of evidence is still not solid enough to recommend routine testing for antibodies targeting PT as shown by a summary of all studies available in the literature (Tables 4 and 5).

### New assays for anti-β_2_GPI antibodies: the anti-domain antibodies

β_2_GPI is a relatively large plasma glycoprotein of 70 kDa with good immunogenic properties. Thus, it is not surprising that APS patients can produce antibodies against several epitopes of the molecule as demonstrated using different experimental approaches. Although the epitope specificity using linear peptides was not originally reported to be associated with specific clinical manifestations of the syndrome, more recently a close association between anti-domain (D)I reactivity and vascular events has been suggested [67].

The reactivity against DI of β_2_GPI was described for the first time in 2002 but its importance was revealed when a two-step technique was used to characterize β_2_GPI-dependent aPLs [68]. Specifically, β_2_GPI coated on hydrophilic but not hydrophobic microtiter plates displays conformational changes that expose DI to the surface, making it more accessible for autoantibody binding. Anti-β_2_GPI antibodies with DI specificity were found in the majority of APS patients and were significantly associated with LA and vascular thrombosis (mostly venous) [69]. Only in a subsequent multicenter study were they also found to be associated with the obstetric manifestations of the syndrome, although to a lesser extent than with thrombosis [70]. However, some data from the multicenter study are controversial. In fact, no correlation between LA and miscarriages was found, in contrast to several previous publications and the known clinical LA predictive value for miscarriages [17]. High levels of antibodies with comparable specificity and detected by a research ELISA kit have been recently associated with an increased risk for thrombotic events in a prospective cohort of SLE or aPL patients by the same group [71].

Recent studies have demonstrated that patients with multiple positive test results (that is, LA, aCLs and anti-β_2_GPI autoantibodies particularly of the IgG isotype) display a much higher risk for developing clinical complications [17]. In line with the hypothesis that anti-DI IgG may represent a more predictive aPL profile, these patients tend to have a higher prevalence and higher titers of anti-β_2_GPI-DI antibodies [72,73].

Anti-β_2_GPI-DI IgG antibodies have been found as the most prevalent antibodies not only in primary APS with thrombosis but also in primary APS with pure obstetric disease. Comparable positivity rates were detectable in patients with SLE or undifferentiated connective tissue diseases, while antibodies against DIV or DV were less frequent in the same populations [73]. aPL positive asymptomatic carriers display a less polarized profile, suggesting that anti-DI antibodies may cluster in patients with systemic autoimmune diseases [73]. Interestingly, the two techniques for anti-DI antibodies used in the study (that is, standard ELISA and chemiluminescence immunoassay (CIA)) have been reported to display the same specificity but different sensitivities [74].

Additional epidemiological studies apparently support the diagnostic/predictive value of anti-DI antibodies. IgG reacting with β_2_GPI in sera from aPL-positive asymptomatic carriers, individuals with leprosy or children with atopic dermatitis have been shown to preferentially recognize epitopes on DIV or DV [68]. Recent studies have suggested that the ratio between anti-β_2_GPI-DI and anti-β_2_GPI-DIV/V IgG antibody reactivities can provide important information to discriminate between anti-β_2_GPI antibodies linked to an autoimmune disease such as APS and antibodies occurring in association with other pathologies [73]. If confirmed in larger studies, this finding would suggest the use of tests for antibodies against the different domains to discriminate between predictive versus non predictive anti-β_2_GPI antibodies.

In any case, it is difficult to draw definite conclusions on the diagnostic and prognostic value of anti-DI antibodies at this stage. The high prevalence of anti-DI IgG antibodies in patients with medium-high titers of aPLs and multiple positivities in the formal diagnostic tests supports the role of DI as the immunodominant epitope of β_2_GPI. However, a small but consistent proportion of full-blown APS patients have autoantibodies reacting with different epitopes, suggesting that the assay for the whole molecule cannot be substituted yet [73]. The discrepancies in the clinical associations can be related to the different methodologies used. Besides the two-step assay, three ELISA studies and a CIA employing different DI molecules or peptides have been reported [74,75]. Although preliminary data seem to indicate that the solid phase assays by two different ELISAs or CIA are comparable, there is no information on the comparison with the two-step assay and the additional ELISAs. Confirmatory studies using multi-center setups and larger prospective patient cohorts are needed to confirm the data.

The fact that the anti-DI antibodies are directed against the immunodominant epitope of β_2_GPI is supported also by pathogenic studies in animal models. Passive infusion of a synthetic antigenic target peptide DI was shown to protect naïve mice from the thrombogenic effects of human polyclonal aPL IgG fractions [76]. Although the inhibition of thrombus formation as well as expression of adhesion molecule on aortic ECs and tissue factor expression on macrophages were not complete, this finding was thought to represent a proof of concept of the pathogenic role of anti-DI antibodies [76]. More recently, a human monoclonal IgG, specifically reacting with DI, was shown to induce clotting and fetal loss in naïve mice, offering the first direct demonstration of the pathogenic effect of anti-DI antibodies [77]. Interestingly, the anti-DI monoclonal induced clotting via complement activation and only after the concomitant administration of small amounts of lipopolysaccharide. This finding is in line with previous results obtained using polyclonal IgG anti-β_2_GPI fractions from APS patients and further supports the potential pathogenic role of anti-DI antibodies.

The main epitope of DI has been suggested to be a cryptic and conformation-dependent structure involving different residues located in the proximity of the junction between DI and DII. Fine epitope mapping using short synthetic peptide fragments and mutation experiments have demonstrated that the main epitope on β_2_GPI-DI is located around amino acid 40 of the molecule with R39-R43 representing the key constituent of the discontinuous epitope [68]. DI of β_2_GPI is usually hidden, being linked with DII in the circular form of the molecule, the most abundant variant in the circulation. After interaction with anionic PL monolayers or when bound to endotoxin, β_2_GPI is opened and DI can be presented to the afferent limb of the immune system [69]. In other words, it could be speculated that, unlike the other domains, which can induce tolerance at high antigen concentrations, DI does not. So, even a small amount of DI presented to the immune system can break the tolerance and easily induce specific antibodies.

### Antibodies against phosphatidylethanolamine

Phosphatidylethanolamine (PE) is a zwitterionic PL, mainly located in the inner leaflets of biological membranes. Subpopulations of aPE bind to high molecular weight kininogen, leading to the formation of antibody-PE-kininogen trimolecular complexes that enhance thrombin-induced platelet aggregation. PE promotes thrombosis by activating factor X and PT, and works as an anticoagulant potentiating activated protein C activity. The finding that PE in the hexagonal phase inhibits the prolongation of clotting time led to the hypothesis that aPEs might be responsible for the LA phenomenon, despite a lack of a significant association between the two assays [78]. A clear *in vivo* demonstration of the pathogenic role of aPEs in mediating vascular and obstetric events is lacking.

Antibodies against PE have been described in up to 43% of APS patients, a higher positivity rate compared to healthy controls [78]. In particular, aPE prevalence among women experiencing recurrent pregnancy loss (RPL) has been reported to range between 23 and 31.7% [79]. Overall, available evidence on the clinical role of aPEs is inconsistent and comes from a limited number of studies, being flawed by the small sample size and poor ELISA standardization [78]. Therefore, aPE testing is currently not recommended, with these autoantibodies not yielding increased accuracy in diagnosing APS. Nevertheless, some authors have proposed aPEs as serological markers of seronegative APS, a debated nosological entity characterized by a clinical picture highly suggestive of APS despite persistent aPL undetectability. In patients with otherwise unexplained thrombotic events, the prevalence of aPEs was 18% when detected by ELISA, rising to 30.5% when tested using thin-layer immunostaining [78,80]. Further, in a multicenter study on 270 thrombotic patients, 63% of 40 aPE-positive subjects had no additional aPL laboratory tests [78].

### Antibodies against anionic phospholipids other than cardiolipin

The diagnostic and prognostic roles of several autoantibodies targeting negatively charged PLs other than CL have been evaluated, though not extensively, in the setting of APS.

PS, phosphatidylinositol and phosphatidic acid, three anionic PLs found in the inner and outer membranes of most cells, are among the best-characterized antigens. Noteworthy, in the 1980s aCLs were shown to broadly cross-react with antibodies targeting both PS and phosphatidylinositol. The cross-reactivity was mostly supported by the recognition of the complex of β_2_GPI with the different anionic PLs. In fact, being a cationic molecule, β_2_GPI binds efficiently to negatively charged PLs. Hence, the largest part of the cross-reactivity is actually due to the same family of autoantibodies, namely those reacting with β_2_GPI [81]. As a whole, testing for antibodies against PS (aPSs), phosphatidylinositol and phosphatidic acid does not improve the likelihood of diagnosing APS compared with criteria tests, being therefore not recommended in international guidelines [1].

Nevertheless, aPSs were reported to be promising, with a particular relevance in obstetric APS. In a study on 872 women with RPL, aPSs were the only detectable aPLs in 3.6% of subjects [82]. However, contradictory data have recently emerged: in one study, aPSs were not related to RPL, while other authors identified IgG but not IgM aPSs as associated with RPL. On the other hand, there is no evidence for an association between aPSs and vascular events [82].

Two murine monoclonal antibodies targeting PS (one reacting with both CL and PS and one with PS only) inhibited the development and invasion by trophoblasts, decreased human chorionic gonadotropin levels and retarded syncytiotrophoblast formation. Unfortunately there is no information whether they recognized β_2_GPI or not [82]. Conversely, the β_2_GPI dependence was shown to be important in another study in which active immunization with β_2_GPI-dependent polyclonal human IgG but not IgM aPSs induced fetal resorption via the production of murine β_2_GPI-dependent IgG aPSs [83]. Discrepancies across available studies justify why APS criteria do not include aPS assays among laboratory tools.

A novel ELISA kit detecting antibodies against a mixture of negatively charged PLs comprising PS, phosphatidic acid and β_2_GPI (APhL) has been recently introduced to the market. This commercial immunoassay has been suggested to overcome the issue of the low specificity of aCLs, which are frequently detected in infectious conditions such as chronic hepatitis C, leprosy, syphilis, and parvovirus B19 infection among others. Furthermore, the APhL assay displays higher positive and negative predictive values for APS diagnosis compared to two commercially available aCL assays [84]. In a cohort of 158 SLE patients, multivariate analysis revealed an association between APhL and thrombotic events, particularly arterial [85].

### Antibodies against vimentin

Proteomic analysis of endothelial-surface membrane proteins in sera from patients with so-called seronegative APS led to the identification of vimentin as a strong autoantigen. Vimentin, a ubiquitous cytoskeleton intermediate filament protein, has been shown to bind CL *in vitro*. In one cohort of patients, antibodies against vimentin/CL were described in 55% of seronegative APS and 92% of full-blown APS patients [82]. However, antibodies against vimentin/CL have also been reported in aPL-negative SLE and rheumatoid arthritis subjects without any clinical manifestation suggestive of APS, thus weakening the specificity of such a diagnostic marker [82].

### Annexins: annexin A5 resistance assay and autoantibodies against annexin A5 and annexin A2

Annexins are a family of ubiquitous calcium-dependent PL-binding proteins. Annexin (Ann)A5 is a potent anticoagulant protein mainly found in trophoblasts and vascular ECs. Upon binding to anionic PLs, it undergoes oligomerization to form a protective shield against coagulation enzymes. β_2_GPI-dependent aPLs have been shown to interfere with the protective binding of AnnA5 to the endothelium, hence leading to thrombosis. A novel two-stage coagulation assay to establish the AnnA5 resistance has been developed; patients with coagulation time lower than the reference are considered AnnA5-resistant [81,82]. Data from five studies revealed that 52% of APS patients were AnnA5-resistant, in comparison with 2 to 5% of controls and seronegative subjects [81]. Resistance to AnnA5 anticoagulant activity was found to inversely correlate with titers of IgG antibodies targeting DI in both thrombotic and obstetric APS [81]. Future studies will assess whether this functional test may allow the identification of specific subsets of pathogenic anti-β_2_GPI antibodies. The clinical significance of serum autoantibodies against AnnA5 has also been investigated: in one study, no association was reported between these autoantibodies and vascular events, while inconsistencies emerged across different studies in obstetric APS [81,82].

AnnA2, a cofactor for plasmin generation and cell-surface localization of fibrinolytic activity, has been identified as a receptor mediating β_2_GPI binding to ECs. Autoantibodies against AnnA2 have been demonstrated to exert a prothrombotic activity by activating ECs, inducing tissue factor expression and blocking tissue plasminogen activator-induced plasminogen activation *in vitro*. A high prevalence of AnnA2 antibodies has been described in patients with APS, but also in some other autoimmune conditions, thus lowering the specificity of this biomarker [86].

## More than just autoantibodies

The presence of aPLs, even if persistent over time, does not explain the full spectrum of APS. For example, a comparable aPL profile can be associated with vascular but not obstetric manifestations and, in some cases, women with APS and previous miscarriages do not display any vascular events [1,4]. In other words, autoantibodies with the same autoantigen specificity and titers have been associated with different clinical pictures and found to support diverse pathogenic mechanisms in experimental models [16].

β_2_GPI-dependent aPL IgG fractions were reported to affect signaling pathways in monocytes and trophoblast cell lines in different ways depending on whether they were obtained from patients with vascular thrombosis or from women with aPL-related miscarriages only [87]. Hence, the eventual clinical picture has been linked to autoantibodies with the same antigen specificity but different biological effects: induction of a pro-thrombotic and inflammatory phenotype in monocytes by β_2_GPI-dependent IgG antibodies from vascular APS patients and inhibition of trophoblast development by β_2_GPI-dependent IgG antibodies from obstetric APS patients [87].

It is still unclear whether these different effects can be related to diverse IgG epitope specificity. The use of these IgG fractions in animal models of aPL-induced thrombosis or fetal loss could further support such an elegant hypothesis *in vivo*.

Vascular and the obstetric APS have been suggested to represent two different variants of the syndrome [88]. The most striking difference is represented by the need of a second hit for triggering thrombosis in naive animals passively infused with human aPLs, while this is not apparently required in models of fetal loss. In fact, the infusion of aPL IgG fractions in pregnant naive mice can itself induce fetal loss and growth retardation. It has been recently demonstrated that β_2_GPI displays a peculiar tissue distribution in resting naive animals, being detectable only at the level of uterine endothelium but not in other vascularized tissues [89]. So the presence of anti-β_2_GPI antibodies can affect pregnancy outcome in resting animals but it does not trigger any vascular thrombosis. Animal pre-treatment with small amounts of lipopolysaccharide may induce the presence of β_2_GPI in vascularized tissues and only then can aPLs react with the target, activate the complement cascade and induce thrombosis [89]. Accordingly, it has been suggested that the modulation of β_2_GPI tissue distribution by inflammatory stimuli may represent an additional variable able to affect the ability of the antibodies to induce the vascular manifestations of the syndrome [16,89].

Post-transcriptional modifications of β_2_GPI, such as oxidation, have been shown to affect autoantibody binding [90]. For example, autoimmune anti-β_2_GPI IgG antibodies react more strongly with plates coated with oxidized β_2_GPI than antibodies obtained after active immunization in naive animals. Moreover, plasma levels of oxidized β_2_GPI have been found to be increased in sera of APS patients. Altogether, these findings suggest that increased levels of post-transcriptionally modified β_2_GPI and the higher antibody reactivity against the modified molecule may affect the biological consequences of aPL binding [90].

**Figure 1:**
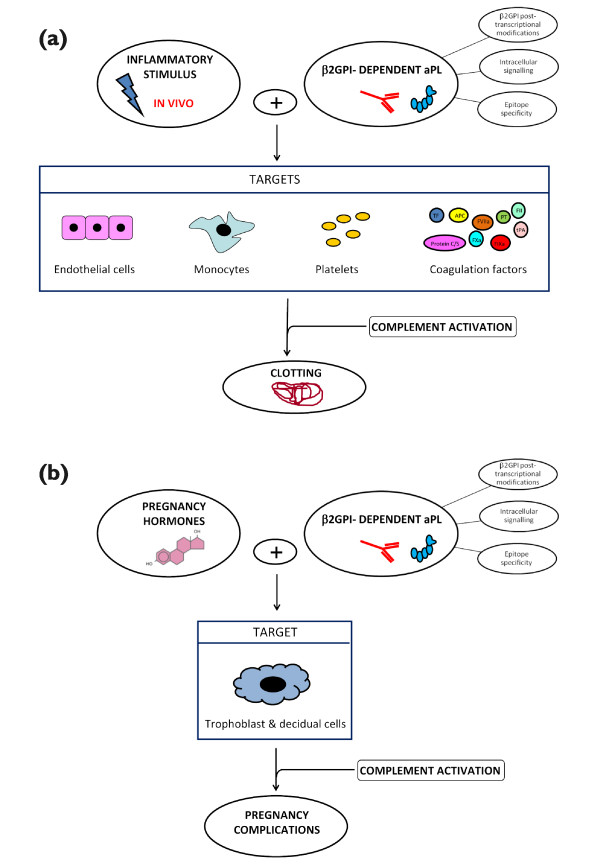
**Schematic views of anti-phospholipid syndrome pathogenic mechanisms. (a)** Vascular anti-phospholipid syndrome (APS). Anti-phospholipid antibodies (aPLs) may target different cell types and soluble coagulations factors. Pathogenic aPLs are beta_2_ glycoprotein I (β_2_GPI)-dependent and activate complement after an inflammatory stimulus (second hit). Additional variables may affect aPL pathogenicity, such as the ability of antibodies to modulate different cell signaling and to display diverse epitope specificity and reactivity with modified β_2_GPI. **(b)** Obstetric APS. β_2_GPI-dependent aPLs may target trophoblast and decidual cells. β_2_GPI can be present at the uterine level even in non-pregnant animals and it binds to trophoblast cells (syncytiotrophoblasts). A second hit is not apparently required, and female hormones or the pregnancy itself may be the equivalent of the second hit described for the vascular manifestations. As in vascular APS, the ability of antibodies to modulate different cell signaling and to display diverse epitope specificity and reactivity with modified β_2_GPI may be additional variables that can affect aPL pathogenicity. APC, activated protein C; C?S, Protein C/S; FII, Factor II; FIXa, Factor IXa, FVIIa, Factor VIIa; FXa, Factor Xa; PT, prothrombin; TF, tissue factor; tPA, tissue plasminogen factor.

In addition, aPLs can be detected in so-called asymptomatic positive carriers who display the persistent presence of medium to high levels of antibodies but in whom no clinical events can be documented. It has been suggested that the second hit cannot take place in these subjects or that their antibodies display different antigen specificity. In line with the last hypothesis, the epitope specificity of the β_2_GPI-dependent IgG antibodies in these subjects was found to be more frequently directed against DIV or DV than against DI as in full-blown APS sera [68,74]. Since the DI epitope is available for the autoantibodies on the open molecule only (for example, when bound to anionic PL monolayers), it has been hypothesized that only these antibodies may be pathogenic. Figure 1 presents a schematic view of the above discussed pathogenic mechanisms.

## Conclusion

The clinical spectrum of APS is more polymorphic than it was thought in the past, making the syndrome much closer to a systemic autoimmune disease. Additional laboratory tests have been proposed in order to improve diagnostic and predictive power, but promising findings have been reported only for anti-PS/PT and anti-DI antibodies.

aPLs play a major pathogenic role in inducing clinical manifestations; however, there is growing evidence that inflammatory stimuli are pivotal for triggering thrombosis, while tissue distribution of the major antigenic target (β_2_GPI) as well as its post-transcriptional modifications or the fine epitope specificity of anti-β_2_GPI antibodies may influence the type of clinical events or even their occurrence.

## Abbreviations

β2GPI: beta_2_ glycoprotein I; aCL: Anti-cardiolipin antibody; Ann: Annexin; aPE: Anti-phosphatidylethanolamine antibody; aPL: Anti-phospholipid antibody; aPS: Anti-phosphatidylserine antibody; APS: Anti-phospholipid syndrome; aPT: Anti-prothrombin antibody; CIA: Chemiluminescence immunoassay; CL: Cardiolipin; D: Domain; EC: Endothelial cell; ELISA: Enzyme-linked immunosorbent assay; Ig: Immunoglobulin; LA: Lupus anticoagulant; PE: Phosphatidylethanolamine; PL: Phospholipid; PS: Phosphatidylserine; PT: Prothrombin; RPL: Recurrent pregnancy loss; SLE: Systemic lupus erythematosus.

## Competing interests

The authors declare that they have no competing interests.
